# The Inactivation of LPS Biosynthesis Genes in *E. coli* Cells Leads to Oxidative Stress

**DOI:** 10.3390/cells11172667

**Published:** 2022-08-27

**Authors:** Tatiana A. Seregina, Irina Yu. Petrushanko, Rustem S. Shakulov, Pavel I. Zaripov, Alexander A. Makarov, Vladimir A. Mitkevich, Alexander S. Mironov

**Affiliations:** Engelhardt Institute of Molecular Biology, Russian Academy of Science, 119991 Moscow, Russia

**Keywords:** LPS mutants, oxidative stress, supersensitivity, antibiotics, reactive oxygen species, reactive sulfur species, thiols

## Abstract

Impaired lipopolysaccharide biosynthesis in Gram-negative bacteria results in the “deep rough” phenotype, which is characterized by increased sensitivity of cells to various hydrophobic compounds, including antibiotics novobiocin, actinomycin D, erythromycin, etc. The present study showed that *E. coli* mutants carrying deletions of the ADP-heptose biosynthesis genes became hypersensitive to a wide range of antibacterial drugs: DNA gyrase inhibitors, protein biosynthesis inhibitors (aminoglycosides, tetracycline), RNA polymerase inhibitors (rifampicin), and β-lactams (carbenicillin). In addition, it was found that inactivation of the *gmhA*, *hldE*, *rfaD*, and *waaC* genes led to dramatic changes in the redox status of cells: a decrease in the pool of reducing NADPH and ATP equivalents, the concentration of intracellular cysteine, a change in thiol homeostasis, and a deficiency in the formation of hydrogen sulfide. In “deep rough” mutants, intensive formation of reactive oxygen species was observed, which, along with a lack of reducing agents, such as reactive sulfur species or NADPH, leads to oxidative stress and an increase in the number of dead cells in the population. Within the framework of modern ideas about the role of oxidative stress as a universal mechanism of the bactericidal action of antibiotics, inhibition of the enzymes of ADP-heptose biosynthesis is a promising direction for increasing the effectiveness of existing antibiotics and solving the problem of multidrug resistance.

## 1. Introduction

**Figure 1:**
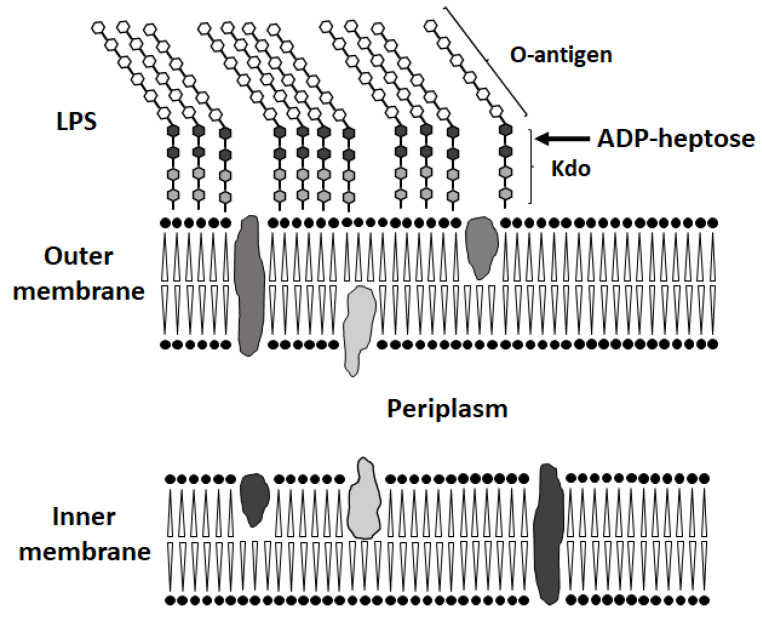
Cell wall structure of gram-negative bacteria. ADP-heptose attaches O-antigen polysaccharide chains to the inner core (Kdo) [4]. The “deep-rough” mutant LPS structure is unstable due to the lack of negatively charged phosphate groups on heptose residues, which normally stabilize the structure through the interaction with positive ions. Furthermore, the “deep-rough” phenotype leads to increased susceptibility to hydrophobic compounds [5].

At present, a large number of studies are devoted to solving the problem of antibiotic resistance to bacterial infections. The most effective direction of this work is not the creation of new antibacterial drugs, but the search for cellular targets, exposure to which would lead to a significant increase in the sensitivity of bacteria to antibiotics, widely used in modern regimens for the treatment of clinically significant infections. Previously, it was described that the disruption of biosynthesis and the assembly of lipopolysaccharide (LPS) components of the outer membrane of Gram-negative bacteria, due to mutations in the *rfa-*operon (“deep-rough” mutants), led to an increase in the content of lipids in LPS as well as a simultaneous decrease in proteins. As a result, there was an increase in membrane permeability for hydrophobic substances and an enhancement in cell sensitivity to various hydrophobic compounds, including antibiotics novobiocin, actinomycin D, erythromycin, etc. [1,2]. The *rfaD*, *waaF*, and *waaC* genes are part of the first of three *rfa*-operons and encode proteins involved in the biosynthesis and transfer of activated heptose residues to the LPS inner core [3]. The *gmhA*, *gmhB*, and *hldE* genes are not included in the *rfa*-operon; their protein products are involved in the isomerization of sedoheptulose-7P and its subsequent activation reactions, resulting in the formation of ADP-glycero-manno-heptose, which, with the participation of the WaaC and WaaF proteins, is attached to the inner core (Kdo) LPS (Figure 1).

**Figure 2:**
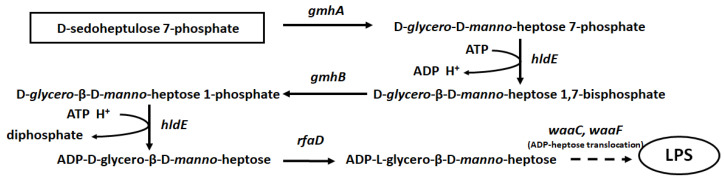
Biosynthesis of ADP-heptose from sedoheptulose 7-phosphate and its translocation to LPS. Disruption of any step in this pathway disturbs the incorporation of the heptose residues in the LPS structure.

The main intracellular source of sedoheptulose in most organisms is the pentose phosphate pathway (PPP) [6]. Sedoheptulose is formed in the reversible branch of PPP by transketolase from two pentose molecules or in the opposite direction by transaldolase from erythrose and fructose. In *E. coli* cells, sedoheptulose-7P is attached to the backbone of LPS (2-keto-3-deoxyoctanate, Kdo) after activation in the form of ADP-D-glycero-β-D-*manno*-heptose. This process involves the *gmhA*, *gmhB*, *hldE* genes, and the *raf*-operon genes *rfaD* (or *gmhD*), *waaC* (*rfaF*), and *waaF* (*rfaC*) (Figure 2) [7,8]. ADP-heptose biosynthetic enzymes are quite conserved among a large spectrum of Gram-negative bacteria of clinical significance [8,9].

A detailed study of mutants with a violation of different stages of LPS synthesis has not been carried out, just as the supersensitivity mechanism of these mutants to various antibiotics remains unclear.

The interest in a more detailed study of LPS synthesis mutants is primarily due to practical considerations. Increased sensitivity to antibiotics makes them promising targets for the search for small-molecule inhibitors which can be used as new antibacterial drugs that enhance the action of traditional antibiotics. In this study, the effect of deletion of the genes *gmhA*, *hldE*, *rfaD*, *gmhB, waaC,* and *waaF* on the redox status of cells, their production of hydrogen sulfide, cell viability, and cellular sensitivity to different antibiotics was evaluated.

## 2. Materials and Methods

### 2.1. Strains and Growth Conditions

*E. coli* strains used in the present study are listed in Table 1. The sequences of the studied genes were downloaded from the Ecocyc database (ecocyc.org). Construction of *gmhA, hldE, gmhB, rfaD, waaC*, and *waaF* deletion strains of *E. coli* was produced according to the method of Datsenko and Wanner [10]. The primers are listed in Table 2. When necessary, the kanamycin drug resistance marker was excised from strains using the FLP activity of pMW int-xis-ts, followed by loss of the plasmid at the nonpermissive temperature. All mutations were verified by PCR and gel analysis. Luria-Bertani (LB) complete medium was used for the general cultivation of *E. coli*. To obtain strains, antibiotics were added at 30 μg/mL (for chloramphenicol) and 100 μg/mL (for ampicillin). A solid medium was prepared using 1.5% agar. All reagents used in the work produced by Sigma-Aldrich, St. Louis, MO, USA, unless otherwise stated.

### 2.2. Determination of Sensitivity to Antibiotics

Overnight cultures were inoculated into a LB broth and grown at 37 °C to ~2 × 10^7^ cells per 1 mL. Cell suspensions were aligned according to optical density, and a series of 6 consecutive tenfold dilutions was prepared. Then the cells were placed on solid media containing various concentrations of antibiotics. To determine the viability of bacterial cells, suspensions were incubated in the presence of lethal doses of antibiotics and then plated on solid media for counting colony-forming units (CFU) [11].

### 2.3. Determination of Viability and Redox Status of Cells Using Flow Cytometry

Cells were grown in a complete medium to an optical density of 0.4 and then washed twice with phosphate-buffered saline (1xPBS), centrifuged, the supernatant was removed, and cells were resuspended in 100 μL of PBS. Cell parameters were analyzed using flow cytometry on a BD LCR Fortessa flow cytometer (Becton Dickinson, Franklin Lakes, NJ, USA). The cell population for analysis was selected according to the parameters of forward (FSC) and side scattering (SSC), which characterize the size and granularity of cells.

The percentage of dead cells in the cell population was assessed using propidium iodide (Ex/Em = 535/617 nm, Sigma-Aldrich, St. Louis, MO, USA), which was added to the cells at a concentration of 10 μg/mL per minute before the start of the analysis. Propidium iodide penetrates the cells with a damaged membrane and, after binding to DNA, has a bright fluorescence in the red region of the spectrum. The redox status of *E. coli* cells was assessed by the level of reactive oxygen species (ROS) and intracellular thiols, a significant part of which is intracellular reduced glutathione (GSH). The ROS level was assessed using the dye Dihydrorhodamine 123 (DHR123) (Ex/Em = 507/525 nm, ThermoFisher Scientific, Waltham, MA, USA) [12], which was added to the cells to a final concentration of 7.5 µM, and the cells were incubated for an hour in the dark at 37 °C. The levels of intracellular thiols were assessed using ThiolTracker Violet dye (Ex/Em = 405/526 nm, ThermoFisher Scientific) [13], which was added at a concentration of 10 μM, after which the cells were incubated for one hour in the dark at 37 °C. Specific levels of superoxide anion were evaluated using Dihydroethidium (DHE) (Ex/Em = 518/606 nm, ThermoFisher Scientific) as described earlier [14]. Cells were incubated with 20 µM Dihydroethidium for 1 h in the dark at 37 °C. These parameters were evaluated in the cells with intact membranes, which were not propidium iodide stained. Each value is the mean of at least three independent experiments with triplicate samples ± SD.

### 2.4. Measurement of NADPH

Cells were grown in 10 mL of LB medium at 37 °C to an optical density of 0.4 and collected by centrifugation. The concentration of reductive NADPH equivalents was determined using the High Sensitivity NADPH Quantitation Fluorometric Assay Kit (Sigma-Aldrich, St. Louis, MO, USA) on a Tecan Spark tablet reader. The obtained values were related to the optical density of the culture. Data were obtained from three independent experiments.

### 2.5. Measurement of ATP

The amount of ATP in cells was determined by the luminescent method using the ATP Detection Assay Kit (Abcam Laboratories, Chicago, IL, USA) on a Tecan Spark tablet reader. One hundred microliters of overnight culture were inoculated with 10 mL of fresh LB medium and grown to an optical density of 0.4. Cells were destroyed by ultrasound. Debris was removed by centrifugation. The resulting extracts were transferred to a plate with the reaction mixture, and the luminescence was immediately measured at a wavelength of 535 nm. Three independent repeats were prepared for each sample.

### 2.6. H_2_S Detection

To detect H_2_S production in wild-type (wt) and mutant cells, the lead acetate detection method was used [15]. Paper strips saturated by 2% of Pb(Ac)_2_ were affixed to the inner wall of a cultural tube, above the level of the liquid culture of wild-type or mutant bacteria. Overnight cultures were diluted at 1:500 in LB and incubated for 16–18 h at 37 °C with aeration. Stained paper strips were scanned and quantified with an Alpha Imager (Imgen Technologies, Alexandria, WA, USA). The results were normalized per ODs.

### 2.7. Determination of Intracellular Cysteine

Intracellular cysteine was determined in cell extracts by the method of Gaitonde [16]. Bacterial cultures were grown in 50 mL LB medium to OD600 0.4. Cells were harvested by centrifugation and suspended in 20 mM EDTA. After that, the cells were destroyed by sonication. Proteins were precipitated by the addition of 5 M HClO_4_. The suspension was centrifuged, and the pH of the supernatant was adjusted to 8.5 by adding KOH. Potassium perchlorate was removed by centrifugation. Samples were treated with 50 mM DTT. The reaction mixture consisted of 500 µL of the sample and 500 µL of Gaitonde’s reagent (250 mg ninhydrin, 4 mL HCl, and 16 mL glacial acetic acid). A calibration curve was built using the known concentrations of cysteine. The assigned values were related to the OD_600_ of the culture. Data were obtained from three independent experiments.

### 2.8. Statistical Analysis

The data are shown as mean ± standard deviation measures from triplicate values obtained from 3–4 independent experiments. The statistical difference between experimental groups was analyzed by one-way ANOVA with Tukey correction for multiple comparisons. Probability values (*p*) less than 0.05 were considered significant. Statistical analysis was performed using the GraphPad Prism 9.1.2 software (GraphPad Software Inc, San Diego, CA, USA).

## 3. Results

### 3.1. Oxidative Stress and Antibiotic Sensitivity

**Figure 3:**
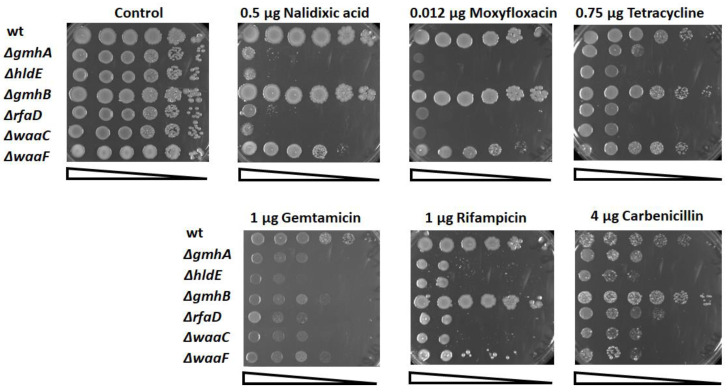
Determination of the sensitivity of strains containing deletions in the *gmhA, hldE*, *gmhB*, *rfaD*, *waaC*, and *waaF* genes to various groups of antibiotics. Cells were spotted on LB agar plates in serial 10-fold dilutions and incubated at 37 °C for 24 h.

Previously, it was shown that Gram-negative bacteria with impaired LPS synthesis were extremely sensitive to various hydrophobic bactericidal agents [17]. During the study, we tested antibacterial drugs with various mechanisms of action: DNA gyrase inhibitors (nalidixic acid, moxifloxacin), protein biosynthesis inhibitors (gentamicin, tetracycline), RNA polymerase inhibitors (rifampicin), and β-lactams (carbenicillin). The sensitivity of the studied mutants was determined by applying a series of dilutions on solid media containing various concentrations of antibiotics (Figure 3). 

Deletions in the *gmhA, hldE*, *rfaD*, and *waaC* genes lead to a dramatic increase in sensitivity (Figure 3).

**Figure 4:**
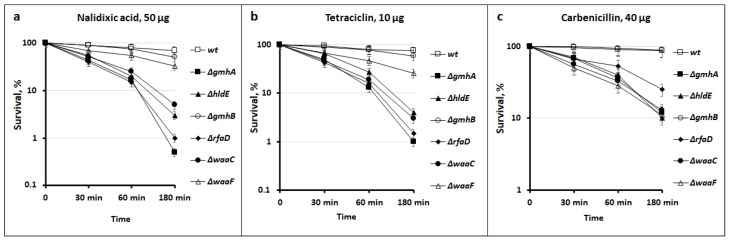
Survival of *E. coli* MG1655 strains containing knockouts for ADP-heptose activation genes (*gmhA*, *hldE*, *gmhB*, *rfaD*, *waaC*, and *waaF*). Representative curves show the effect of deficiency ADP-heptose biosynthesis on antibiotic-mediated killing. Mean values ± SD from at least three independent experiments are shown.

In addition, survival analysis was performed for each mutant on nalidixic acid, tetracycline, and carbenicillin. Bacterial cells were incubated in the presence of lethal doses (means the minimum bactericidal concentration) of antibiotics and plated on a solid medium for further CFU counting (Figure 4). Survival was expressed as a percentage of untreated control.

Deletions of *gmhA, hldE, rfaD*, and *waaC* sensitize *E. coli* cells to all the studied classes of antibiotics, while ∆*waaF* exhibits very moderate sensitivity, and the ∆*gmhB* mutant does not differ from wild-type bacteria.

**Figure 5:**
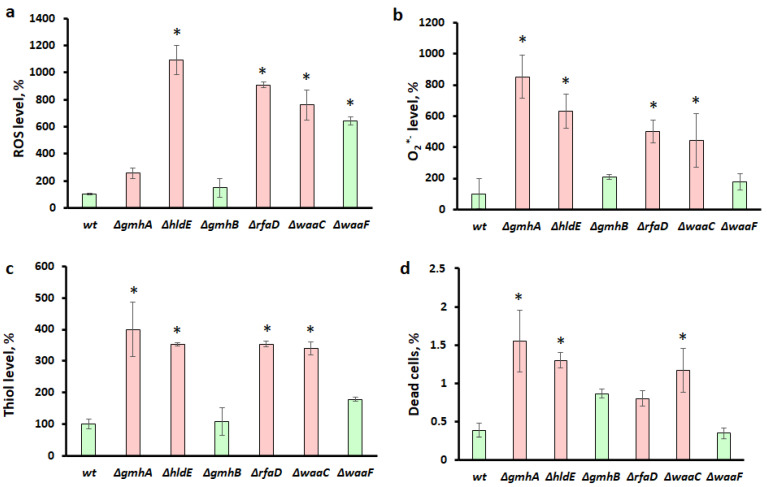
Changes in the level of intracellular ROS, O_2_^*−^ the level of thiols, and the percentage of dead cells in the population of *E. coli* cells with deletions of the *gmhA, hldE, gmhB, rfaD, waaC, waaF* genes. The level of ROS (**a**) was assessed using the dye DHR123; the O_2_^*−^ level (**b**) with DHE dye; the thiols level (**c**) with ThiolTracker Violet; the percentage of dead cells (**d**) was estimated using propidium iodide. Parameters for cells with *gmhA*, *hldE*, *rfaD*, and *waaC* deletions that result in a dramatic increase in antibiotic susceptibility in *E. coli* cells are shown in red, while parameters for *wt*, *gmhB,* and *waaF* are shown in green. Mean values ± SD from at least three independent experiments are shown. *—*p* < 0.05, compared to the wild-type cells.

There is ample evidence for the involvement of ROS in antibiotic toxicity by inducing oxidative damage to DNA and proteins [18]. Evaluation of reactive oxygen species (ROS) in cells showed that deletions of the *hldE* gene induced maximal oxidative stress (Figure 5a). The level of ROS increased by more than 10 times. Deletions of the *gmhA*, *rfaD, waaC,* and *waaF* genes led to a less pronounced increase in ROS by 3, 9, 8, and 6 times, correspondingly. The ROS level in the ∆*gmhB* mutant did not differ from the wild-type bacteria. It was found that the O_2_^*−^ level for ∆*hldE, ∆rfaD*, and ∆*waaC* correlated with the ROS level (Figure 5b). Unexpectedly, the O_2_^*−^ level was maximal for the *gmhA* mutant, which did not have such a high ROS level as other mutants. At the same time, O_2_^*−^ levels in ∆*waaF* and ∆*gmhB* were similar and close to values for the wild-type cells. Deletions of *gmhA,* *hldE, rfaD*, and *waaC* led to a significant (more than three-fold) increase in the thiol group level in *E. coli*. In cells with a deletion of *waaF*, the increase in thiols was less pronounced (less than 2 times). At the same time, the amounts of thiols in the ∆*gmhB* mutant did not differ from wild-type bacteria (Figure 5c). The percentage of dead cells in the population was also maximal in the case of a deletion of *gmhA*. In the cell populations with deletions of *hldE,* and *waaC*, the percentages of dead cells were slightly lower than at ∆*gmhA*, and in the case of ∆*gmhB, ∆rfaD,* and ∆*waaF*, it statistically did not differ from the wild-type bacteria.

The data obtained demonstrate a clear correlation between the amount of ROS, dead cells, an increase in reduced thiols, and sensitivity to antibiotics in “deep rough” mutants. It is interesting, that the mutants showing increased sensitivity to antibiotics (red color in the histogram) differ sharply in the redox status of the cells from the wild-type bacteria (Figure 5). 

### 3.2. Reducing NADPH Equivalents and the ATP Pool

The cofactors ATP, NADH, and NADPH constitute the core of an intricate biochemical network, the operation of which must be clearly regulated in changing environmental conditions. Reducing equivalents of NADPH are the main electron donors in the redox reactions and are essential for many anabolic reactions to occur. One of the main sources of NADPH formation in a bacterial cell is the oxidative branch of the pentose phosphate shunt. In addition, it is in this metabolic pathway that the precursor of ADP-heptose, sedoheptulose 7-phosphate, is formed.

**Figure 6:**
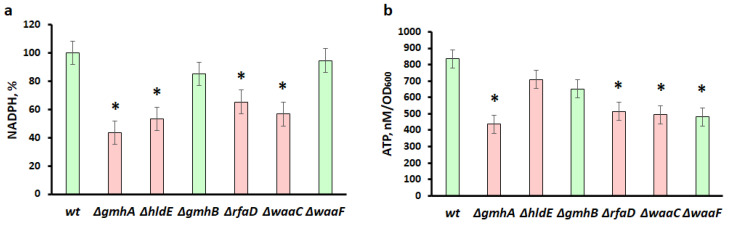
Changes in the intracellular content of NADPH and ATP in the population of *E. coli* cells with deletions of the *gmhA, hldE, gmhB, rfaD, waaC, waaF* genes. (**a**) Changes in the intracellular NADPH level. (**b**) Changes in the intracellular ATP level. Parameters for cells with *gmhA*, *hldE*, *rfaD*, and *waaC* deletions that result in a dramatic increase in antibiotic susceptibility in *E. coli* cells are shown in red, while parameters for *wt*, *gmhB,* and *waaF* are shown in green. Mean values ± SD from at least three independent experiments are shown. *—*p* < 0.05, compared to the wild-type cells.

Against the background of deletions of *gmhA,* *hldE*, *rfaD*, and *waaC*, there was a significant decrease in the level of NADPH compared to the wild-type strain (Figure 6a). The effect of *gmhB* and *waaF* deletions on NADPH generation was much weaker and it statistically did not differ from the wild-type bacteria. It can be assumed that NADPH deficiency occurs as a result of its consumption to extinguish oxidative stress, restore glutathione, and support other protective mechanisms.

A significant decrease in NADPH in ADP-heptose of deficient strains is possibly associated with the suppression of oxidative stress that occurred against the background of changes in cell wall permeability (Figure 6a). 

ATP deficiency can be a consequence of both a malfunction of the respiratory chain and other metabolic processes associated with its consumption (Figure 6b). It was shown that the greatest decrease in the ATP pool occurs in mutants containing deletions of the *gmhA, rfaD*, *waaC*, and *waaF* genes (Figure 6b).

### 3.3. H_2_S Generation and Cysteine Deficiency

Hydrogen sulfide in bacterial cells has an extremely important physiological function and is a component of the most ancient redox-regulatory system involving active forms of sulfur. Previously, it was shown that H_2_S could protect bacteria from antibiotics and oxidative stress [19,20]. It was found that bacterial cells with a low level of hydrogen sulfide generation were more sensitive to the action of antibiotics and oxidizing agents such as hydrogen peroxide. The main source of H_2_S generation in *E. coli* cells is the pathway of cysteine degradation; therefore, hydrogen sulfide can also be considered an indicator of the intensity of cysteine metabolism.

**Figure 7:**
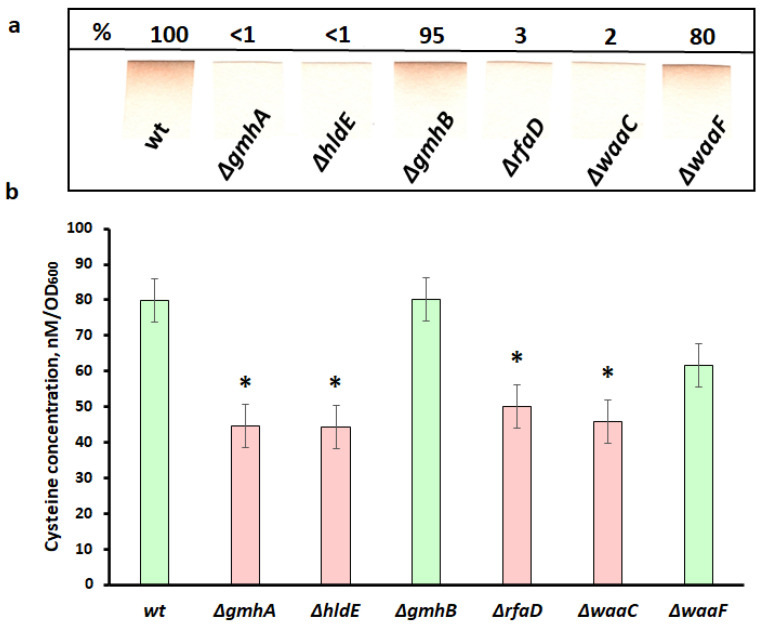
Formation of hydrogen sulfide and intracellular concentration of cysteine in the population of *E. coli* cells with deletions of the *gmhA, hldE, gmhB, rfaD, waaC, waaF* genes and wild-type cells. (**a**) Formation of H_2_S by *E. coli* cells. Pb-acetate-soaked paper strips show a brown colour of PbS as a result of the reaction with gaseous H_2_S exiting liquid bacterial cultures (**b**) Intracellular concentration of cysteine. Parameters for cells with *gmhA*, *hldE*, *rfaD*, and *waaC* deletions that result in a dramatic increase in antibiotic susceptibility in *E. coli* cells are shown in red, while parameters for *wt*, *gmhB,* and *waaF* are shown in green. Mean values ± SD from at least three independent experiments are shown. *—*p* < 0.05, compared to the wild-type cells.

During the experiments, it was found that mutants carrying deletions of the *gmhA*, *hldE*, *rfaD*, and *waaC* genes stopped generating hydrogen sulfide, which may indicate a change in the metabolism of cysteine, as the main precursor of H_2_S biosynthesis, while the *gmhB* and *waaF* mutants practically did not differ from the control in this parameter (Figure 7a).

Previously, it was shown that the level of endogenous H_2_S generation directly depended on the intracellular content of cysteine [20], as it is a precursor in the hydrogen sulfide biosynthetic pathway. The main role in the generation of H_2_S in *E. coli* cells, as was shown earlier, is played by the enzyme 3-mercaptopyruvate sulfo-transferase (3-MST) encoded by the *mstA* gene [21].

To test the assumption that the decrease in the level of H_2_S generation in the *gmhA*, *hldE*, *rfaD*, and *waaC* mutants is due to a low level of intracellular cysteine content, we performed a quantitative determination of this amino acid in living cells (Figure 7b). The intracellular concentration of cysteine was determined spectrophotometrically using the Gaitonde reagent [16].

As follows from the data presented in Figure 7, in all mutants, with the exception of *gmhB*, a marked decrease in the cytoplasmic pool of cysteine was observed. These data correlate well with the level of hydrogen sulfide generation in these mutants. Thus, in the strains where H_2_S generation was practically absent (*gmhA, hldE, rfaD, waaC*) (Figure 7a), the intracellular cysteine concentration was significantly reduced (Figure 7b).

## 4. Discussion

The results obtained reveal that strong oxidative stress develops in *E. coli* cells against the background of impaired LPS biosynthesis. The results of this work clearly indicate that the level of sensitivity of different mutants to antibiotics correlates well with a decrease in the intracellular content of NADPH, ATP, and cysteine, the generation of hydrogen sulfide, thiol levels, and an increase in the intracellular ROS. In the strains ∆*gmhA*, ∆*hldE*, ∆*rfaD,* and ∆*waaC* with high sensitivity, there is a dramatic increase in ROS, one type of which is superoxide (Figure 5). On the other hand, the mutant ∆*waaF* is significantly less sensitive to the tested antibiotics, which can be partly explained by the emergence of compensatory mechanisms (fitness costs) [22,23]. The mutant ∆*gmhB* is practically the same as the wild-type strain. In these strains, changes in redox status and other measured parameters were not as considerable. This is due to the fact that ∆*gmhB* does not lead to a complete disruption of ADP-heptose synthesis due to the presence of homologous enzymes. The *gmhB* gene product (D-glycero-β-D-manno-heptose-1,7-bisphosphate 7-phosphatase) is an enzyme belonging to the phosphatase class. Several similar enzymes are present in *E. coli* cells, for example, the imidazoleglycerol-phosphate dehydratase/histidinol-phosphatase HisB involved in histidine biosynthesis, according to the literature [1], can replace the *gmhB* deletion defect. In addition, there are phosphatases involved in the central carbon metabolism, the activity of which is critical for cell viability; therefore, it is not possible to completely block this stage of ADP-heptose biosynthesis [24,25]. However, a tendency to slightly increase in dead cells in the ∆*gmhB* and ∆*rfaD* populations was detected. Furthermore, the outer membrane integrity is monitored by the envelope stress response system, which initiates the activity of genes and enzymes involved in membrane remodulation. The loss of GmhB activates periplasmic serine endoprotease DegP production, resulting in porin OmpF degradation [26]. Changes in the number of porins in the outer membrane can affect the osmotic resistance of cells.

An interesting fact is that strains with impaired ADP-heptose synthesis actively produce colanic acid, which forms a capsule and compensates for the defect in the barrier function of the impaired cell wall [27,28,29]. In contrast with other mutants, ∆*waaF* secretes large amounts of colanic acid, this biosynthetic pathway can deplete ATP [29]. However, the release of colanic acid into the growth medium leads to an increase in viscosity and deterioration of gas exchange [30], which can lead to the activation of the respiratory chain, the main source of reactive oxygen species. Apparently, the increased generation of ROS in the ∆*waaF* strain can be explained by changes in the functioning of the respiratory chain [31]. On the other hand, keeping enough reduction equivalents in ∆*waaF* (Figure 6 and Figure 7) will compensate for oxidative stress [32] and prevent an increase in the number of dead cells (Figure 5d).

Furthermore, an increase in the number of reduced thiol groups also indicates significant changes in the metabolism of reactive sulfur species in LPS mutants (∆*gmhA*, ∆*hldE*, ∆*rfaD,* and ∆*waaC*). Cysteine, along with hydrogen sulfide and other low-molecular-weight thiols form an important regulatory system that maintains the redox balance of the bacterial cell and its resistance to stress. Cysteine, being in the external environment mainly in the oxidized form (cystine), is transported into the cell, where it undergoes rapid recovery. Cystine is present in bacterial cells in trace amounts that cannot be detected [33]. Currently, cysteine is considered one of the main protectors against ROS in the periplasm of gram-negative bacteria [34,35]. On the other hand, the concentration of cysteine in the cell cytoplasm is strictly controlled, since an excess of cysteine can promote the genotoxic Fenton reaction [35,36]. As a reason for the increased sensitivity of mutants to antibiotics, one can observe a decrease in the intracellular concentration of cysteine (Figure 7). It can be hypothesized that the disruption of LPS integrity leads to the uncontrolled outflow of cysteine from the cell, and also, possibly, to the difficulty in the functioning of the L-cysteine/cystine shuttle system. A decrease in the intracellular concentration of cysteine, in turn, leads to a decrease in the level of H_2_S generation (Figure 7), which, as was shown earlier, protects cells from oxidative stress and the action of antibiotics [19,20]. It is important to note that in the experiments on measuring the generation of H_2_S in mutants, a complete LB medium was used. Under these conditions, an important contribution to the intracellular pool of cysteine is made by its transport in the form of cystine, which is a component of the LB environment and is carried out with the participation of specific transporters *tcyP* and *tcyJ* [34]. This explanation is supported by experiments to determine the intracellular content of cysteine in selected LPS mutants. Moreover, the development of oxidative stress in ADP-heptose deficient cells can promote the activation of metabolic fluxes in order to compensate for the increased consumption of reducing agents. The formation and reduction/oxidation of NADP/NADPH is in strict relationship with energy metabolism. One of the nonspecific effects of antibiotics is an increase in cellular respiration, which is directly related to the synthesis of ATP [31]. This process requires reductive NAD(H) equivalents, which in turn are linked to the NADP(H) pool via the enzymes pyridine nucleotide transhydrogenases PntAB and UdhA [32]. Previous research suggests that the metabolic state of bacteria influences their susceptibility to antibiotics [37]. According to our data, a significant reduction in the pool of NADPH and ATP is observed in ∆*gmhA*, ∆*rfaD*, and ∆*waaC* mutants, which indicates an imbalance in central metabolism (Figure 6). It is possible that the deletion of *hldE* leads to the conservation of ATP compared to other deep-rough mutants, since this enzyme catalyses the ATP-dependent addition of phosphate in the first position to D-glycero-*manno*-heptose 7-phosphate and subsequent transformation of D-glycero-β-D-manno-heptose 1-phosphate into ADP-L-glycero-β-D-*manno*-heptose.

Thus, a change in the normal structure of the LPS of the cell wall of gram-negative bacteria not only leads to a violation of its barrier functions but also significantly affects the key protective mechanisms. Redox imbalance of deep-rough mutants deprives cells of the opportunity to form an adequate response to oxidative stress caused by the antibacterial drugs. The most significant changes in redox homeostasis in *E. coli* cells occur when the *gmhA*, *hldE*, *rfaD*, and *waaC* genes are deleted (Figure 5, Figure 6 and Figure 7) and these cells show a dramatic increase in antibiotic sensitivity (Figure 3 and Figure 4). In the future, it would be interesting to analyze the homology of these enzymes among clinically significant pathogens, as well as to determine their influence on antibiotic susceptibility.

Previously, attempts were made to create inhibitors of the LPS biosynthesis enzyme [3,7,9,38]. The idea of (should these words “The idea of” be removed?) inactivating the biosynthesis of ADP-heptose is considered a way to create antivirulent adjuvants, since this component of LPS is responsible for the formation of biofilms and, in fact, is an endotoxin [38]. After nearly a decade of inactivity, new data emerges now. Recent research has revealed that ADP-heptose biosynthesis affects antibiotic susceptibility in *Helicobacter pylori* and *Mycobacterium tuberculosis* [39,40]. Based on previous and our data we consider it promising to continue this work, since the inactivation of these enzymes leads to an unprecedented increase in cell sensitivity, and will make it possible to overcome the problems of antibiotic resistance of gram-negative pathogens.

## Figures and Tables

**Table 1:** Genotype of obtained mutants with deletions of LPS biosynthesis genes.

Genotype	Nature of Mutation	Reference
wt	F^-^ wild type	Laboratory collection
∆*gmhA*	Deletion of the *gmhA* D-sedoheptulose 7-phosphate isomerase	This work
∆*hldE*	Deletion of the *hldE* gene encoding heptose-7-phosphate kinase	“
∆*gmhB*	Deletion of the *gmhB* gene encoding	“
	D,D-heptose 1,7-bisphosphate phosphatase	
∆*rfaD*	Deletion of the *rfaD* gene encoding	“
	ADP-L-glycero-D-mannoheptose-6-epimerase	
∆*waaC*	Deletion of the *waaC* gene encoding	“
	ADP-heptose:LPS heptosyltransferase I	
∆*waaF*	Deletion of the *waaF* gene encoding	“
	ADP-heptose:LPS heptosyltransferase II	

**Table 2:** Primers for obtaining deletions of LPS biosynthesis genes.

Name	Sequence
GmhA1	5′–atg-tac-cag-gat-ctt-att-cgt-acc-gaa-ctg-aac-gac-gct-caa-gtt-agt-ata-aaa-aag-ct–3′
GmhA2	5′–tta-ctt-aac-cat-ctc-ttt-ttc-aat-taa-ctg-gat-cag-tga-agc-ctg-ctt-ttt-tat-act-aag–3′
GmhA3	5′–ccg-tac-ttc-tcg-ctt-ttg-gc–3′
GmhA4	5′–taa-gac-gcg-tca-gca-tcg-ca–3′
HldE1	5′–atg-aaa-gta-acg-ctg-cca-gag-ttt-gaa-cgt-gca-ggc-gct-caa-gtt-agt-ata-aaa-aag-ct–3′
HldE2	5′–tta-gcc-ttt-ttt-atc-ctg-ttg-gat-ctt-ctt-gat-gat-tga-agc-ctg-ctt-ttt-tat-act-aag–3′
HldE3	5′–ggt-gga-aga-atg-aag-tat-gg–3′
HldE4	5′–gtt-gaa-aaa-aca-aca-gcg-tca–3′
GmhB1	5′–gtg-gcg-aag-agc-gta-ccc-gca-att-ttt-ctt-gac-cgc-gct-caa-gtt-agt-ata-aaa-aag-ct–3′
GmhB2	5′–tca-ttg-tgc-cgg-ttt-ttg-ctg-ctt-ttt-tat-cgc-ttg-tga-agc-ctg-ctt-ttt-tat-act-aag–3′
GmhB3	5′–ttc-ttg-cag-gtc-gaa-aca-tg–3′
GmhB4	5′–c-tca-gga-aga-caa-gcg-gaa–3′
RfaD1	5′–atg-atc-atc-gtt-acc-ggc-ggc-gcg-ggc-ttt-atc-ggc-gct-caa-gtt-agt-ata-aaa-aag-ct–3′
RfaD2	5′–tta-tgc-gtc-gcg-att-cag-cca-ggc-cat-gta-ttc-cgt-tga-agc-ctg-ctt-ttt-tat-act-aag–3′
RfaD3	5′–atg-att-aca-gac-att-cgt-gtc–3′
RfaD4	5′–ga-ctt-tgc-gac-atc-atc-atg–3′
WaaC1	5′–atg-cgg-gtt-ttg-atc-gtt-aaa-aca-tcg-tcg-atg-ggc-gct-caa-gtt-agt-ata-aaa-aag-ct–3′
WaaC2	5′–tta-taa-tga-tga-taa-ctt-ttc-caa-aac-tgc-ttg-act-tga-agc-ctg-ctt-ttt-tat-act-aag–3′
WaaC3	5′–gcg-tac-tgg-aag-aac-tca-ac–3′
WaaC4	5′–gat-ttc-aga-gtg-taa-ggt-ttc–3′
WaaF1	5′–atg-aaa-ata-ctg-gtg-atc-ggc-ccg-tct-tgg-gtt-ggc-gct-caa-gtt-agt-ata-aaa-aag-ct–3′
WaaF2	5′–tca-ggc-ttc-ctc-ttg-taa-caa-tag-cgc-gtt-gag-ttc-tga-agc-ctg-ctt-ttt-tat-act-aag–3′
WaaF3	5′–gtt-gct-gaa-ggt-gta-acg-ga–3′
WaaF4	5′–ggc-aac-gta-tgg-aga-aca-tc–3′

## Data Availability

This study did not report any data.
